# Annotations

**Published:** 1898-08-20

**Authors:** 


### Annotations.

### Conscription.

Every time when, as at present, the air is full of rumours of war, there creeps into public thought, if not into public desire, an anticipation of the time, which perhaps may never come, but is not outside the bounds of possibility, when in Britain, as on the Continent, every man will be called on to spend a year or two in military service. Peace, as we know, is maintained by being prepared for war; and while our main defence must be always, as it is now, our fleet, backed by our standing army, it would be no disadvantage if that, in turn, were backed by a whole nation of trained soldiers. We have a general horror of conscription; but while we may most reasonably deplore the chance of a war that would involve the services of the whole nation in the field, it does not follow that we have any cause to fear, or even to dislike, so much preparation for it as is implied in sending our young men for a year or two to serve in the army. Military drill and military discipline are not in themselves bad things, but good. Young men who, going direct from school to the workshop, or, worse, to the college or the desk, rather weaken than strengthen their constitution at a time when they should be building it up against the strain of life, would be greatly benefited by the compulsory physical culture given by the drill-sergeant. Nor would the moral discipline be bad. It is not a bad thing for a young man at the most conceited and self-sufficient time of his life—when his theories of life have not been put to the test of life itself—to be compelled to yield unquestioning obedience to orders which he does not fully understand the meaning of. "Their's not to reason why; their's but to do or die," may be but a soldier's virtue; but success, even in civil life, is not often won by those who are not willing to do the immediate duty before them without knowing all the reasons that make it imperative. Two years' training in promptitude and accuracy would in many cases amply compensate for the loss of two years of ordinary work. So much for the probable effect on those who were soldiers only under compulsion; but the effect on the regular army would not be less beneficial. At present our army is largely recruited from idlers and ne'er-do-wells, who can find no opening in civil life. This, at least, is the general belief; and, though the idea is not wholly accurate, there is a considerable amount of truth in it. If that be so, we must the more marvel at the deeds, not only of daring, but of endurance, more difficult still, that our soldiers perform; and when we note the many posts of responsibility that old soldiers fill, we must assume that the majority of them leave the army better men than they entered it. But if the discipline of the army be, as it seems to be, a good thing in itself, how much better would the whole tone of the army be, if all men, gentle and simple, had to serve in its ranks. The influence of those of higher ideals would inevitably have a wholesome effect on the others, and the whole tone of army life would improve. Abroad this has proved to be the case, and there is no reason why it should not be the same here. Then "soldier" and "vagabond" would no longer be, as in popular estimation they now too often are, convertible terms; and the soldier, being no longer a dog with a bad name, would not so often deserve hanging.

### The Victims of Typhoid.

Who, for the most part die from typhoid fever? Is it the prosperous middle-class, who live in modern villas, inartistic, perhaps, but certainly sanitary? These occasionally pick up the disease in "health resorts" (falsely so-called), native or foreign, where sea or mountain air, fine scenery, and it may be a mineral well thrown in, are supposed to make up for the lack of those conveniences which the ordinary Briton would never think of doing without at home. But they don't catch typhoid at home. The people in the slums do; but who expects sanitation in the slums? Sanitary officers may do their best; but the people who regard it as a matter of course to throw the household garbage into the gutter, and let excreta lie about the back garden, are there all the time, and the sanitary officer comes only occasionally. It will take generations of education to teach these people that they are committing suicide by their neglect of sanitary precautions; and sanitary science is such a new thing that it has not yet reached them. But one cannot but wonder sometimes if sanitary science has reached the upper classes, the dwellers in those "stately homes of England" which form such a picturesque feature in many of our landscapes? Picturesqueness is a good thing, but it is not quite such a good thing as health, and often the two seem to be incompatible. Doubtless in the manors of our gentry, in the castles of our nobles, you will find all those comforts which custom associates with good sanitation; but it does not always follow that the two are combined. Baths and an abundant supply of drinking water are good, but you want to know where the water comes from; drains are good, but you want to know where the drains go. And in these points many mansions are faulty. In the early days of sanitary science, many things were hid which ought to have been revealed. New drain-pipes were laid, but they ran only into old cesspools, which were built over, and then supposed to be innocuous, while their gases, if not their fluid contents, percolated through the surrounding earth. New extensions are built to old houses, but the foundations rarely go deep enough to unveil the old field drains which have run for hundreds of years, once open but now covered over, near the old walls. New drains may be put in the house, smoke-tested, proved to be completely satisfactory, so far as they go, but the old sewers below escape discovery until some mysterious illness, and perhaps a death or two, give rise to suspicion. It is to these hidden causes that many deaths from typhoid among our upper classes may be traced. An old house, built when sanitation was unknown or—and perhaps this is even more dangerous—when it was in its infancy, is very rarely a healthy one. Probably no one living knows where all its drains find an outlet, and, in consequence, unsuspected poisons filter into the veins of the dwellers, in the midst of the most recent appliances for health and comfort; the moral of which is, not indeed that every house more than twenty years old should be destroyed, but that the owners of old houses should be more careful in putting sanitary conveniences into them and in making sure that all refuse matter is safely conveyed outside, and that they should make a point of knowing where it goes.

